# Synthesis of New *N,N’*-Bis(5-arylidene-4-oxo-4,5-dihydrothiazolin-2-yl)piperazine Derivatives Under Microwave Irradiation and Preliminary Biological Evaluation

**DOI:** 10.3797/scipharm.1206-04

**Published:** 2012-09-16

**Authors:** Wacothon Karime Coulibaly, Ludovic Paquin, Anoubilé Bénié, Yves-Alain Bekro, Emilie Durieux, Laurent Meijer, Rémy Le Guével, Anne Corlu, Jean-Pierre Bazureau

**Affiliations:** 1 Université de Rennes 1, Institut des Sciences Chimiques de Rennes (ISCR), UMR CNRS 6226, groupe Ingénièrie Chimique et Molécules pour le Vivant (ICMV), Bât. 10A, campus de Beaulieu, Avenue du Général Leclerc, CS 74205, 35042 Rennes Cedex, France.; 2 Université d’Abobo-Adjamé, Laboratoire de Chimie Bioorganique et de Subtances Naturelles (LCBSN), BP 802, Abidjan 02, République de la Côte d’Ivoire.; 3 Protein Phosphorylation and Human Disease Group, Station Biologique CNRS, Place G. Tessier, BP 74, 29682 Roscoff, France.; 4 ManRos Therapeutics (From Sea to Pharmacy), Hôtel de Recherche, Centre de Perharidy, 29680 Roscoff, France.; 5 Université de Rennes 1, ImPACcell Platform, IFR 140, Bât. 8, 2 Avenue du Prof. Léon Bernard, CS 34317, 35043 Rennes Cedex, France.

**Keywords:** 5-Arylidene rhodanine, 2,2’-(Piperazine-1,4-diyl)bis(5-benzylidene-1,3-thiazol-4(5*H*)-one), Microwave irradiation, Cytotoxity, Protein kinase, Diamines

## Abstract

New *N,N’*-bis(5-arylidene-4-oxo-4,5-dihydrothiazoline-2-yl)diamine derivatives **5** were prepared in two steps from rhodanine and piperazine, or 1,4-bis(3-amino-propyl)piperazine, under microwave reaction conditions with retention of configuration. Some of these compounds were tested for *in vitro* antiproliferative activities and for their kinase inhibitory potencies towards six kinases (CDK5/p25, GSK3α/β, DYRK1A, DYRK2, CLK1, and CLK2). The compound **5d** showed nanomolar activity towards DYRK1A kinase (IC_50_ = 0.041 μM).

## Introduction

**Fig. 1.**
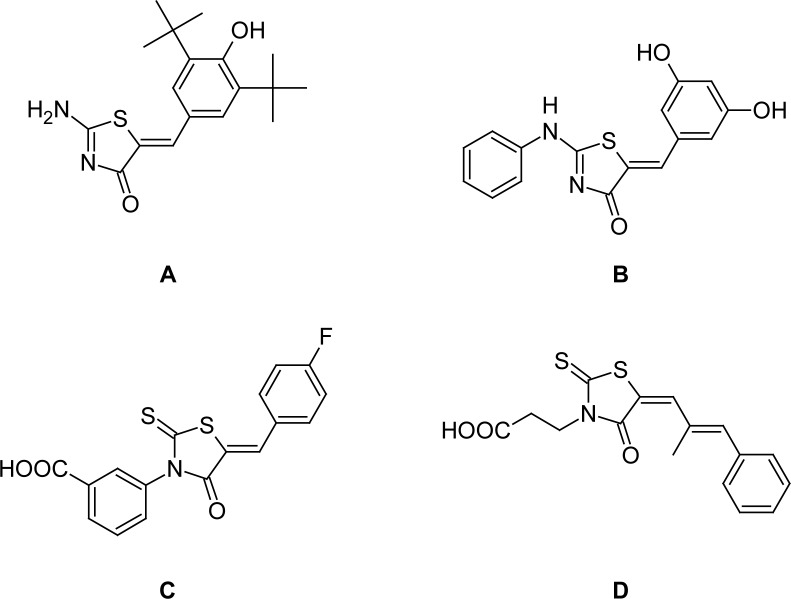
Structures of Darbufelone (**A**), DBPT (**B**), inhibitor of DSP (**C**), and epalrestat (**D**).

The 2-amino-5-arylidene-5*H*-thiazol-4-ones and their 5-arylidene rhodanine precursors are a class of five-membered heterocyclic rings (FMHRs) considered as “privileged scaffolds” in the medicinal chemistry community [1]. Considerable work has been published over decades about their chemistry and biology. A number of compounds containing the 2-amino-5-arylidenethiazol-4(5*H*)-one moiety have been shown to exhibit anti-inflammatory [2], antimicrobial [3], and antitumor [4] effects. Among these compounds, Darbufelone® **A**[5] (Figure 1) is orally active in animal models of inflammation [6] and DBPT **B** is under clinical trials for colon cancer [7]. 5-Arylidene rhodanines have also proven to be attractive for the discovery of new candidates. A series of rhodanine-based hits **C** (Figure 1) were found as potent and selective inhibitors of the “atypical” dual-specificity phosphatase (DSP) family member-JNK-stimulating phophatase-1 (JSP-1). Compounds of this class may be useful for the treatment of inflammatory and proliferative disorders [8]. As the last example, epalrestat **D** reduced the symptoms of diabetic neuropathy [9].

Protein kinases are the enzymes which control the phosphorylation of protein in cellular life [10] which is frequently deregulated in human diseases. For the pharmaceutical industry, the protein kinases represent interesting targets for new therapeutic agents [11] and this interest was boosted by the approval of the first marketed inhibitor Gleevec™ used in myeloid leukemia [12]. Due to the biological activity associated with the 2-amino-thiazolidinone moiety, we decided in this paper to explore the synthesis of *N,N’*-bis(5-arylidene-4-oxo-4,5-dihydrothiazolidin-2-yl)diamines derived from piperazine or 1,4-bis(3-aminopropyl)piperazine as linkers, and to study their effects on cells and protein kinases.

## Results and Discussion

### Chemistry

The strategy used for the synthesis of the symmetric derivatives **5** is outlined in Scheme 1. The reactions have been realized under microwave irradiation [13]. The main benefits of microwave irradiation technology are the significant rate-enhancements and sometimes elevated product yields enabling the rapid synthesis of molecules of potential value in medicinal chemistry [14]. The synthesis started by the solution-phase Knoevenagel condensation of aryl aldehydes **2** and commercial rhodanine **1a**. The expected compounds **3a–c** were prepared in yields ranging from 65 to 88% with a reaction time of 10 min. under microwave irradiation (MWI) at 65°C in the presence of sodium acetate. In a similar approach, the 5-arylidene rhodanine propanoic derivatives **3d–g** were easily synthesized using solvent-less reaction conditions under microwave irradiation (130°C, 10 min.). The geometric double bond of **3** was attributed as being *Z* by the shielding effect of the carbonyl group C-4 on the olefinic proton H-5 (δ 7.5 ppm) in the ^1^H NMR spectra.

Transformation of 5-arylidene rhodanine **3** into 2-amino-5-arylidene-5*H*-thiazol-4-one after addition of a primary amine [15] usually involves activation of the C=S bond of rhodanine via the thioether intermediate that subsequently undergoes a thioalkyl/nitrogen displacement. In order to be able to carry out such sulfur/nitrogen displacement in a faster and more efficient way – avoiding the preparation of the thioether intermediate – we examined the influence of microwave irradiation on the reaction between compound **3** and the symmetric diamino linkers **4a,b**. The experiments revealed that optimal reaction conditions were obtained at 80–120°C after 30 minutes. It is noteworthy that sulfur/nitrogen displacement reactions at 120°C under microwave irradiation have been realized in solution with hexane to avoid decomposition of compound **5**. The desired compounds **5a–e** were prepared in poor to moderate yields (13–26%) and their structures were substantiated by ^1^H, ^13^C NMR, and HRMS analyses and only the more thermo-dynamically stable *Z-*isomers were obtained.

### Biology

To evaluate the potency of the compounds **3a–c**, **5a,** and **5c** for their *in vitro* antiproliferative activities, we used six representative tumor cell lines of liver (Huh7), colon (Caco2, HCT 116), breast (MDA-MB 231), prostate (PC3), lung (NCI), and one normal cell line (fibroblats) and measured survival. Results are reported in Table 1. The 5-arylidene rhodanines **3a–c** and two compounds **5a** and **5c** showed measurable, albeit very poor, cytotoxic activity. No clear tendency for higher activity of these compounds can be deduced from these results. Since the log P values of all tested compounds are in the same range, influences of lipophilicity on cytotoxic activity cannot be deduced as well.

The kinase inhibitory potencies of compounds **3a–c** and **5d** were evaluated as IC_50_ values towards six protein kinases (CDK5/p25, GSK3α/β, DYRK1A, DYRK2, CLK1 and CLK2) and the results are reported in Table 2. The 5-arylidene rhodanines **3a,b** appeared to be inactive towards these six protein kinases, but interesting results were obtained with compounds **5d** and **3c**. The 1,4-bis[(5*Z*)-5-(4-methoxybenzylidene)methylene-4-oxo-4,5-dihydrothiazol-2-yl]piperazine **5b** and the 5-arylidene rhodanine **3c** are very active on DYRK1A [16] and a noteworthy IC_50_ = 41 nM was measured for **5b**. The compound **5d** has also shown submicromolar inhibition potencies towards DYRK2 (IC_50_ = 0.6 μM) and CLK1 (IC_50_ = 0.5 μM). Regarding these results, the potential of the (5*Z*) 5-arylidene-4-oxo-4,5-dihydro-thiazolidine-2-yl moiety appended on 1,4-bis(3-aminopropyl)piperazine **4b** as a linker could be highly interesting in the development of a new class of inhibitors of DYRK1A kinase which is known to be involved in Alzheimer’s disease/Down syndrome [17].

## Conclusion

In conclusion, we worked out a short and practical synthesis under microwave irradiation of *N,N’*-(5-arylidene-4-oxo-4,5-dihydrothiazolidin-2-yl)diamines **5a–e** derived from piperazine **4a**[18, 19] and 1,4-bis(3-aminopropyl)piperazine **4b**. The *in vitro* anti-proliferative activities are extremely weak and this could be due to a lower cellular penetration of compounds **3** and **5** or a lower interaction with the cellular targets. Surprisingly, the compound **5d** has shown nanomolar inhibition potency towards DYRK1A and this interesting inhibition led us to expand our efforts in the synthesis of diversely disubstituted *N,N’*-bispiperazine derivatives with the 5-arylidene thiazolidinone moiety as new potential inhibitors of this kinase. Work is in progress to gain deeper insight into the structure-activity relationships (SAR) of this new interesting class of diamines.

## Experimental

### General

Elemental analysis: Flash EA1112 CHN/O Thermo Electron; HRMS (MS/MS ZABSpec Tof Micromass, EBE TOF geometry, IP 8 eV); NMR: BRUKER AC 300P (^1^H: 300 MHz, ^13^C: 75 MHz); melting points: Leica System Kofler VMHB Melting Point apparatus (not corrected); microwave reactor: Monowave 300 Anton-Paar (850 W), monowave software package.

### General procedure I: Preparation of 5-arylidene-2-thioxo-thiazolidine-4-one 3a–c under microwave irradiation

A mixture of rhodanine **1a** (1 g, 7.5 mmol), aldehyde **2** (8.95–9 mmol), sodium acetate (1.85 g, 22.55 mmol), and methanol (5 ml) was placed in a borosilicate glass vial (10 ml) with a Teflon® magnetic stir bar and sealed with a snap cap. The glass tube was then introduced into the Monowave 300 Anton-Paar microwave cavity (P = 850 Watt) and the stirred mixture was irradiated at 65°C (with a power of 10 Watt) for 30 min. After microwave dielectric heating, the crude reaction mixture was allowed to cool down to room temperature, and then was filtered on a Buchner funnel and the insoluble compound **3** was washed with methanol (2x5 ml). The crude compound **3** was purified by recrystallization from methanol and further dried under high vacuum (10^−2^ Torr) for 1 hour, which gave the desired compound **3** as a powder.

### General procedure II: Preparation of 3-(5-arylidene-4-oxo-2-thioxo-thiazolidine-3-yl)propanoic acid 3d–g under solvent-free microwave irradiation

A mixture of rhodanine **1b** (0.5 g, 2.44 mmol) and aldehyde **2** (2.44 mmol) was placed in a borosilicate glass vial (10 ml) with a Teflon® magnetic stir bar and sealed with a snap cap. The glass tube was then introduced into the Monowave 300 Anton-Paar microwave cavity (P = 850 Watt) and the stirred mixture was irradiated at 130°C for 10 min. After microwave dielectric heating, the crude reaction mixture was allowed to cool down to room temperature and 5 ml of a mixture of ethanol/hexane (1:1) were added directly to the glass vial. The resulting precipitated product **3** was filtered on a Buchner funnel and the insoluble compound **3** was washed with the same mixture (2x5 ml). The crude compound **3** was further dried under high vacuum (10^−2^ Torr) for 1 hour, which gave the desired compound **3** as a powder.

### General procedure III: Synthesis of N,N’-bis(5-arylidene-4-oxo-4,5-dihydro-thiazolidine-2-yl)piperazine derivatives 5a–e under microwave irradiation

A mixture of 5-arylidene-2-thioxo-thiazolidine-4-one **3** (4 mmol), piperazine **4a** (172 mg, 2 mmol) or 1,4-bis(3-aminopropyl)piperazine **4b** (400 mg, 2 mmol) was placed in a borosilicate glass vial (10 ml) with a Teflon® magnetic stir bar and sealed with a snap cap. The glass tube was then introduced into the Monowave 300 Anton-Paar microwave cavity (P = 850 Watt) and the stirred mixture was irradiated at 80–120°C (with a power of 5–200 Watt) for 30 min. After microwave dielectric heating, the crude reaction mixture was allowed to cool down to room temperature and 10 ml of cooled ethanol (4°C) were added directly in the glass vial. The resulting precipitated product **5** was filtered off, washed with 2 × 5 ml of ethanol and dried under high vacuum (10^−2^ Torr) at room temperature for 1 hour. After ^1^H NMR analysis, the product **5** was purified by recrystallization from methanol, which gave the desired compound **3** as a powder.

### (5Z)-5-Benzylidene-2-thioxo-1,3-thiazolidin-4-one (3a)

Prepared following General procedure I from benzaldehyde **2a** (0.95 g, 8.95 mmol) to give 1.08 g (65%) as an orange powder. Mp = 208–210°C. ^1^H NMR (300 MHz, DMSO-*d*_6_) δ (ppm) = 7.44–7.55 (m, 5H, Ar), 7.57 (s, 1H, =CH), 13.12 (br s, 1H, NH). ^13^C NMR (75 MHz, DMSO-*d*_6_) δ (ppm) = 126.88 (C-1), 129.20 (C-2), 130.30 (C-3), 130.37 (C-4), 130.45 (C=, C-5), 133.22 (=CH), 171.28 (C=S, C-2); 196,64 (C=O, C-4). HRMS, *m/z* = 243.9868 found (calculated for C_10_H_7_NONaS_2_, [M+Na]^+^ requires 243.9867). Anal. Calcd. for C_10_H_7_NOS_2_: C, 54.27; H, 3.19. Found: C, 54.23; H, 3.16.

### (5Z)-5-(4-Methoxybenzylidene)-2-thioxo-1,3-thiazolidin-4-one (3b)

Prepared following General procedure I from 4-methoxybenzaldehyde **2b** (1.23 g, 9 mmol) to give 1.36 g (74%) as an orange powder. Mp = 250–252°C. ^1^H NMR (300 MHz, DMSO-*d*_6_) δ (ppm) = 3.82 (s, 3H, OCH_3_), 7.10 (d, *J* = 8.8Hz, 2H, H-3, Ar), 7.55 (d, *J* = 9.1 Hz, 2H, H-2, Ar), 7.57 (s, 1H; =CH), 13.76 (br s, 1H, NH). ^13^C NMR (75 MHz, DMSO-*d*_6_) δ (ppm) = 55.45 (OCH_3_), 114.92 (C-3’), 124.90 (C=, C-5), 126.02 (C-1’), 129.71 (=CH), 132.28 (C-2’), 160.82 (C-4’), 173.024 (C=S, C-2), 197.25 (C=O, C-4). HRMS, *m/z* = 295.9792 found (calculated for C_11_H_9_NO_2_Na_2_S_2_, [M-H+2Na]^+^ requires 295.9792). Anal. Calcd. for C_11_H_9_NO_2_S_2_: C, 52.57; H, 3.61. Found: C, 52.65; H, 3.67.

### (5Z)-5-(1,3-Benzodioxol-5-ylmethylidene)-2-thioxo-1,3-thiazolidin-4-one (3c)

Prepared following General procedure I from piperonaldehyde **2c** (1.35 g, 9 mmol) to give 1.74 g (88%) as an orange powder. Mp > 260°C. ^1^H NMR (300 MHz, DMSO-*d*_6_) δ (ppm) = 6.13 (s, 2H; OCH_2_O), 7.11–7.45 (m, 3H; H-5’, H-6’, Ar), 7.52 (s, 1H, =CH), 7.55 (s, 1H, H-2’; Ar), 13.74 (br s, 1H, NH). ^13^C NMR (75 MHz, DMSO-*d*_6_) δ (ppm) = 102.10 (OCH_2_O), 109.26 (C=, C-5), 109.45 (C-6’), 123.02 (C-1’), 126.65 (C-5’), 127.16 (=CH), 131.81 (C-2’), 148.27 (C-4’), 149.60 (C-3’), 169.58 (C=S, C-2), 195.51 (C=O, C-4). HRMS, *m/z* = 287.9765 found (calculated for C_11_H_7_NO_3_NaS_2_, [M+Na]^+^ requires 287.9765). Anal. Calcd. for C_11_H_7_NO_3_S_2_: C, 49.80; H, 2.66. Found: C, 49.91; H, 2.69.

### 3-[(5Z)-5-Benzylidene-4-oxo-2-thioxo-1,3-thiazolidin-3-yl]propanoic acid (3d)

Prepared following General procedure II from benzaldehyde **2a** (0.256 g, 2.44 mmol) to give **3d** in 60% yield as a brown powder. Mp = 224–226°C. ^1^H NMR (300 MHz, DMSO-*d*_6_) δ (ppm) = 2.33 (m, 2H, CH_2_), 4.15 (m, 2H, CH_2_), 7.51–7.65 (m, 5H, H-2, H-3, H-4, Ar), 7.80 (s, 1H, =CH), 13.16 (br s, 1H, OH). ^13^C NMR (75 MHz, DMSO-*d*_6_) δ (ppm) = 33.30 (CH_2_), 41.78 (CH_2_), 122.6 (C=, C-5), 129.46 (C-2’), 130.57 (C-3’), 130.84 (C-4’), 132.63 (=CH), 133.02 (C-1), 166.76 (C=O, C-4), 171.94 (CO_2_H), 193.15 (C=S, C-2). HRMS, *m/z* = 337.9904 found (calculated for C_13_H_10_NO_3_Na_2_S_2_, [M-H+2Na]^+^ requires 337.9898).

### 3-[(5Z)-5-(4-Methoxybenzylidene)-4-oxo-2-thioxo-1,3-thiazolidin-3-yl]propanoic acid (3e)

Prepared following General procedure II from 4-methoxybenzaldehyde **2b** (0.332 g, 2.44 mmol) to give **3e** in 60% as a brown powder. Mp > 260°C. ^1^H NMR (300 MHz, DMSO-*d*_6_) δ (ppm) = 2.24 (t, 2H, J = 8.2 Hz, CH_2_), 3.83 (s, 3H, OCH_3_), 4.12 (t, 2H, J = 8.2 Hz, CH_2_), 7.10 (d, 2H, J = 8. 8 Hz, H-3, Ar), 7.64 (d, 2H, J = 8.8 Hz, H-2, Ar), 7.86 (s, 1H, =CH), 13.26 (br s, 1H, OH). ^13^C NMR (75 MHz, DMSO-*d*_6_) δ (ppm) = 34.11 (CH_2_), 42.20 (CH_2_), 55.56 (OCH_3_), 115.11 (C-3’), 119.26 (C=, C-5), 125.58 (C-1’); 129.90 (=CH), 132.84 (C-2’), 161.41 (C-4’), 166.86 (C=O, C-4), 171.98 (CO_2_H), 192.86 (C=S, C-2). HRMS, *m/z* = 346.0185 found (calculated for C_14_H_13_NO_4_NaS_2_, [M+Na]^+^ requires 346.0184).

### 3-[(5Z)-5-(1,3-Benzodioxol-5-ylmethylidene)-4-oxo-2-thioxo-1,3-thiazolidin-3-yl]propanoic acid (3f)

Prepared following General procedure II from piperonal **2c** (0.366 g, 2.44 mmol) to give **3f** in 63% as a brown powder. Mp = 220–224°C. ^1^H NMR (300 MHz, DMSO-*d*_6_) δ (ppm) = 2.61 (t, 2H, J = 3.9 Hz, CH_2_), 4.21 (t, 2H, J = 4.7 Hz, CH_2_), 6.15 (s, 2H, OCH_2_O), 7.09–7.23 (m, 3H, H-2, H-5, H-6, Ar), 7.74 (s, 1H, =CH), 12.50 (br s, 1H, OH). ^13^C NMR (75 MHz, DMSO-*d*_6_) δ (ppm) = 30.74 (CH_2_), 40.30 (CH_2_), 102.21 (OCH_2_O), 109.35 (C-2’), 109.61 (C-5’), 119.71 (C=, C-5), 126.98 (C-6’), 127.14 (C-1’), 133.21 (=CH), 148.35 (C-4’), 149.88 (C-3’), 166.67 (C=O, C-4), 171.72 (CO_2_H), 192.97 (C=S, C-2). HRMS, *m/z* = 346.0185 found (calculated for C_14_H_13_NO_4_NaS_2_, [M+Na]^+^ requires 346.0184).

### 3-[(5Z)-5-(2,3-Dihydro-1,4-benzodioxin-6-ylmethylidene)-4-oxo-2-thioxo-1,3-thiazolidin-3-yl]propanoic acid (3g)

Prepared following General procedure II from 1,4-benzodioxane-6-carboxaldehyde (**2d**) (0.401 g, 2.44 mmol) to give **3g** in 54% yield as a brown powder. Mp = 226–228°C. ^1^H NMR (300 MHz, DMSO-*d*_6_) δ (ppm) = 2.55 (t, 2H, J = 7.3 Hz, CH_2_), 4.18 (t, 2H, J = 7.4 Hz, CH_2_), 4.30 (m, 4H, OCH_2_CH_2_O), 7.02 (d, 1H, J = 8.6 Hz, H-5, Ar), 7.12 (d, 1H, J = 2.2 Hz, H-6, Ar), 7.15 (s, 1H, H-2, Ar), 7.70 (s, 1H; =CH), 12.46 (br s, 1H, OH). ^13^C NMR (75 MHz, DMSO-*d*_6_) δ (ppm) = 31.30 (CH_2_), 40.30 (CH_2_), 64.01 (OCH_2_CH_2_O), 118.22 (C-2’), 119.47 (C-5’), 119.79 (C=, C-5), 124.46 (C-6’), 126.31 (C-1’), 132.96 (=CH), 143.82 (C-4’), 146.25 (C-3’); 166.67 (C=O, C-4), 171.80 (CO_2_H), 192.93 (C=S, C-2). HRMS, *m/z* = 374.0138 found (calculated for C_15_H_13_NO_5_NaS_2_, [M+Na]^+^ requires 374.0133).

### (5Z,5’Z)-2,2’-(Piperazine-1,4-diyl)bis(5-benzylidene-1,3-thiazol-4(5H)-one) (1,4-Bis[(5Z)-5-benzylidene-4-oxo-4,5-dihydro-1,3-thiazol-2-yl]piperazine, 5a)

Prepared following General procedure III from 5-benzylidene-2-thioxo-thiazolidin-4-one (**3a**) (885 mg, 4 mmol) and piperazine **4a** (172 mg, 2 mmol) in hexane (2 ml) at 120°C to give **5a** in 13% yield as a brown powder. Mp > 260°C. ^1^H NMR (300 MHz, DMSO-*d*_6_) δ (ppm) = 3.34 (br s, 8H, N(CH_2_)_4_N), 7.28–7.7 (m, 12H, H-2’, H-3’, H-4’, Ar, =CH). ^13^C NMR (75 MHz, DMSO-*d*_6_) δ (ppm) = 42.45 (N(CH_2_)_4_N), 129.08 (C-2’), 129.16 (=CH), 129.22 (C=), 129.55 (C-3’), 129.82 (C-4’), 129.94 (C-1’); 130.41 (C=N), 159.56 (C=O). HRMS, *m/z* = 483.0918 found (calculated for C_24_H_20_N_4_O_2_NaS_2_, [M+Na]^+^ requires 483.0925). Anal. Calcd. for C_24_H_20_N_4_O_2_S_2_: C, 62.59; H, 4.38. Found: C, 62.56; H, 4.41.

### (5Z,5’Z)-2,2’-(Piperazine-1,4-diyl)bis[5-(4-methoxybenzylidene)-1,3-thiazol-4(5H)-one] (1,4-Bis[(5Z)-5-(4-methoxybenzylidene)-4-oxo-4,5-dihydro-1,3-thiazol-2-yl]piperazine, 5b)

Prepared following General procedure III from 5-(4-methoxybenzylidene)-2-thioxothiazolidin-4-one (**3b**) (1.005 g, 4 mmol) and piperazine **4a** (172 mg, 2 mmol) at 80°C to give **5b** in 36% yield as a yellow powder. Mp > 260°C. ^1^H NMR (300 MHz, DMSO-*d*_6_) δ (ppm) = 2.99 (s, 8H, N(CH_2_)_4_N), 3.79 (s, 6H, OCH_3_), 7.04 (d, J = 5 Hz, 4H, H-3’, Ar), 7.16 (s, 2H, =CH), 7.43 (d, J = 8.7 Hz, 4H, H-2’, Ar). ^13^C NMR (75 MHz, DMSO-*d*_6_) δ (ppm) = 42.99 (N(CH_2_)_4_N), 55.27 (OCH_3_), 114.54 (C-3’), 114.73 (C-1’), 124.13 (=CH), 127.55 (C=, C-5), 131.29 (C-2’), 131.38 (C-4’), 132.14 (C=N, C-2), 159.56 (C=O, C-4). HRMS, *m/z* = 521.1317 found (calculated for C_26_H_25_N_4_O_4_S_2_, [M+H]^+^ requires 521.1310). Anal. Calcd. for C_26_H_24_N_4_O_4_S_2_: C, 59.98; H, 4.65. Found: C, 59.87; H, 4.64.

### (5Z,5’Z)-2,2’-(Piperazine-1,4-diyl)bis[5-(1,3-benzodioxol-5-ylmethylene)-1,3-thiazol-4(5H)-one] (1,4-Bis[(5Z)-5-(1,3-benzodioxol-5-ylmethylene)-4-oxo-4,5-dihydro-1,3-thiazol-2-yl]piperazine, 5c)

Prepared following General procedure III from 5-(1,3-benzodioxol-5-ylmethylidene)-2-thioxothiazolidin-4-one (**3c**) (1.061 g, 4 mmol) and piperazine **4a** (172 mg, 2 mmol) in hexane (2 ml) at 120°C to give **5a** in 15% yield as a brown powder. Mp > 260°C. ^1^H NMR (300 MHz, DMSO-*d*_6_) δ (ppm) = 3.02 (s, 8H, N(CH_2_)_4_N), 6.11 (s, 4H, OCH_2_O), 7.02–7.18 (m, 6H, H-2’, H-5’, H-6’, Ar), 7.58 (s, 2H, =CH). ^13^C NMR (75 MHz, DMSO-*d*_6_) δ (ppm) = 52.72 (N(CH_2_)_4_N), 101.88 (OCH_2_O), 108.71 (C-2’), 125.13 (C-5’), 126.04 (C=, C-5), 127.86 (C-1’), 130.41 (C-6’), 144.21 (=CH), 146.02 (C=N, C-2), 148.11 (C-4’), 148.78 (C-3’), 182.22 (C=O, C-4). HRMS, *m/z* = 571.0726 found (calculated for C_26_H_20_N_4_O_6_NaS_2_, [M+Na]^+^ requires 571.0722). Anal. Calcd. for C_26_H_20_N_4_O_6_S_2_: C, 56.92; H, 3.67. Found: C, 56.96; H, 3.71.

### (5Z,5’Z)-2,2’-[Piperazine-1,4-diylbis(propane-3,1-diylimino)]bis[5-(4-methoxybenzylidene)-1,3-thiazol-4(5H)-one] (1,4-Bis(3-{[(5Z)-5-(4-methoxybenzylidene)-4-oxo-4,5-dihydro-1,3-thiazol-2-yl]amino}propyl)piperazine, 5d)

Prepared following General procedure III from 5-(4-methoxybenzylidene)-2-thioxothiazolidin-4-one (**3b**) (1.005 g, 4 mmol) and 1,4-bis(3-aminopropyl)piperazine (**4b**) (0.4 g, 2 mmol) in hexane (2 ml) at 120°C to give **5d** in 25% yield as a yellow powder. Mp = 220–222°C. 1H NMR (300 MHz, DMSO-*d*_6_) δ (ppm) = 1.68 (quint, J = 6.9 Hz, 4H, NCH_2_CH_2_CH_2_N(CH_2_)_4_N), 2.37 (t, J = 6.7 Hz; 4H, NCH_2_CH_2_CH_2_N(CH_2_)_4_N), 2.57 (s, 8H, NCH_2_CH_2_CH_2_N(CH_2_)_4_N), 2.84 (t, J = 7.1 Hz, 4H; NCH_2_CH_2_CH_2_N(CH_2_)_4_N), 3.79 (s, 6H, OCH_3_), 7.02 (d, J = 8.8 Hz, 4H, H-3’, Ar), 7.18 (s, 2H; =CH), 7.44 (d, J = 8.8 Hz, 4H, H-2’, Ar). ^13^C NMR (75 MHz, DMSO-*d*_6_) δ (ppm) = 23.39 (NCH_2_CH_2_CH_2_N(CH_2_)_4_N), 37.41 (NCH_2_CH_2_CH_2_N(CH_2_)_4_N), 52.03 (NCH_2_CH_2_CH_2_N(CH_2_)_4_N), 54.42 (NCH_2_CH_2_CH_2_N(CH_2_)_4_N), 55.27 (OCH_3_), 114.58 (C-3’), 125.04 (=CH), 127.28 (C-1’), 130.99 (C=), 131.44 (C-2’), 159.77 (C-4’), 201.61 (C=N, C-2), 206.74 (C=O, C-4). HRMS, *m/z* = 635.2396 found (calculated for C_32_H_39_N_6_O_4_S_2_, [M+H]^+^ requires 635.1819). Anal. Calcd. for C_32_H_38_N_6_O_4_S_2_: C, 60.54; H, 6.03. Found: C, 60.57; H, 6.07.

### (5Z,5’Z)-2,2’-[Piperazine-1,4-diylbis(propane-3,1-diylimino)]bis[5-(1,3-benzodioxol-5-ylmethylene)-1,3-thiazol-4(5H)-one] (1,4-Bis(3-{[(5Z)-5-(1,3-benzodioxol-5-ylmethylene)-4-oxo-4,5-dihydro-1,3-thiazol-2-yl]amino}propyl)piperazine, 5e)

Prepared following General procedure III from 5-(1,3-benzodioxol-5-ylmethylidene)-2-thioxothiazolidin-4-one (**3c**) (1.061 g, 4 mmol) and 1,4-bis(3-aminopropyl)piperazine (**4b**) (0.4 g, 2 mmol) in hexane (2 ml) at 120°C to give **5e** in 15% yield as a brown powder. Mp = 222–224°C. ^1^H NMR (300 MHz, DMSO-*d*_6_) δ (ppm) = 1,69 (quint, J = 6.9 Hz, 4H, NCH_2_CH_2_CH_2_N(CH_2_)_4_N), 2.37 (t, J = 6.6 Hz, 4H, NCH_2_CH_2_CH_2_N(CH_2_)_4_N), 2.37 (s, 8H, NCH_2_CH_2_CH_2_N(CH_2_)_4_N), 2.83 (t, J = 7.1 Hz, 4H, NCH_2_CH_2_CH_2_N(CH_2_)_4_N), 6.07 (s, 4H, OCH_2_O), 7.02 (m, 6H, H-2’, H-5’, H-6’, Ar), 7.14 (s, 2H, =CH). ^13^C NMR (75 MHz, DMSO-*d*_6_) δ (ppm) = 23.47 (NCH_2_CH_2_CH_2_N(CH_2_)_4_N), 37.45 (NCH_2_CH_2_CH_2_N(CH_2_)_4_N), 52.22 (NCH_2_CH_2_CH_2_N(CH_2_)_4_N), 54.54 (NCH_2_CH_2_CH_2_N(CH_2_)_4_N), 101.52 (OCH_2_O), 108.74 (C-2’), 108.82 (C-5’), 124.21 (C-6’), 124.84 (=CH), 147.72 (C-4’), 147.89 (C-3’), 182.66 (C=, C-5), 202.14 (C=N, C-2), 206.72 (C=O, C-4). HRMS, *m/z* = 663.1981 found (calculated for C_32_H_35_N_6_O_6_S_2_, [M+H]^+^ requires 663.7790). Anal. Calcd. for C_32_H_34_N_6_O_6_S_2_: C, 57.99; H, 5.17. Found: C, 57.92; H, 5.16.

### In vitro kinase inhibition assays

*Buffer A*: 10 mM MgCl_2_, 1 mM EGTA, 1 mM DTT, 25 mM Tris-HCl pH 7.5, 50 μg heparin/ml. *Buffer C*: 60 mM ß-glycerophosphate, 15 mM *p*-nitrophenylphosphate, 25 mM Mops (pH 7.2), 5 mM EGTA, 15 mM MgCl_2_, 1 mM DTT, 1 mM sodium vanadate, 1 mM phenylphosphate. Kinase activities were assayed in Buffer A or C, at 30°C, at a final ATP concentration of 15 μM. Blank values were subtracted and activities expressed in % of the maximal activity, i.e. in the absence of inhibitors. Controls were performed with appropriate dilutions of DMSO. The kinase peptide substrates were obtained from Millegen (Labege, France). *DYRK1A* and *DYRK2* (human, recombinant, expressed in *E. coli* as a GST fusion protein) were purified by affinity chromatography on glutathione-agarose and assayed in buffer A (+ 0.5 mg BSA /ml) using Woodtide (KKISGRLSPIMTEQ) (1.5 µg/assay) as a substrate, in the presence of 15 µM [γ-^33^P] ATP (3,000 Ci/mmol; 10 mCi/ml) in a final volume of 30 μl. After 30 min incubation at 30°C, the reaction was stopped by harvesting onto P81 phosphocellulose papers (Whatman) using a FilterMate harvester (Packard) and were washed in 1% phosphoric acid. Scintillation fluid was added and the radioactivity was measured in a Packard counter. *CLK1* and *CLK3* (human, recombinant, expressed in *E. coli* as GST fusion proteins) were assayed in buffer A (+ 0.15 mg BSA /ml) with RS peptide (GRSRSRSRSRSR) (1μg/assay). *CDK5/p25* (human, recombinant) was prepared as previously described [26]. Its kinase activity was assayed in buffer C, with 1 μg histone H1 /ml. *GSK-3α/β* (porcine brain, native) was assayed in Buffer A and using a GSK-3 specific substrate (GS-1: YRRAAVPPSPSLSRHSSPHQSpEDEEE) (pS stands for phosphorylated serine) [20].

### Cell culture and survival assays

Skin diploid fibroblastic cells were provided by BIOPREDIC International Company (Rennes, France). Caco-2 cells and Huh7 cells were obtained from the ECAC collection. Cells were grown according to ECAC recommendations. The RLEC-F1 clone was derived from an established rat biliary epithelial cell line as previously described [21]. The toxicity test of the compounds on these cells was as follows: 4 × 10^3^ cells were seeded in 96 multiwell plates and left for 24 h for attachment, spreading, and growing. Then they were exposed for 48 h to increasing concentrations of the compounds, ranging from 0.1 to 25 μL in a final volume of 80 μL of culture medium. They were then fixed with 4% paraform-aldehyde solution and the nuclei were stained with Hoechst 3342 and counted using automated imaging analysis (Simple PCI software).

## Figures and Tables

**Sch. 1.**
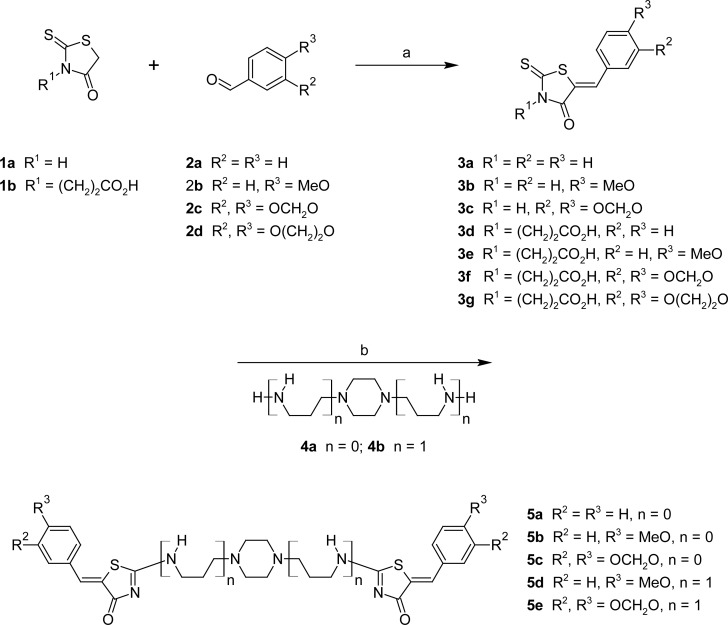
a) for **3a–c**: MeOH, AcONa 3 eq., MWI, 65°C, 10 min. and for **3d–g**: MWI, 130°C, 10 min. b) MWI, 80–120°C, 30 min.

**Tab. 1.** Cell effects of the products and calculated partition coefficients log P (calculated with Chem Draw Pro, Cambridge Soft)

**Cpd.**	**Cell lines IC_50_ (μM)**							
	**Huh7**	**Caco2**	**MDA-MB 231**	**HCT 116**	**PC3**	**NCI**	**Fibroblats**	**Log P_calc._**
**3a**	> 25	> 25	> 25	> 25	> 25	> 25	> 25	2.0
**3b**	> 25	> 25	> 25	> 25	> 25	> 25	> 25	1.9
**3c**	> 25	> 25	> 25	> 25	> 25	> 25	> 25	1.8
**5a**	> 25	25	> 25	> 25	> 25	> 25	> 25	4.8
**5c**	> 25	20	> 25	> 25	40	> 25	40	5.3
Roscovitine	10	10	15	8	8	20	> 25	–
DMSO	> 25	> 25	> 25	> 25	> 25	> 25	> 25	–

**Tab. 2.** CDK5/p25, GSK3α/β, DYRK1A, DYRK2, CLK1, CLK3 inhibitions.

**Cpd.**	**IC_50_ (μM)**					
	**CDK5/p25**	**GSK3α/β**	**DYRK1A**	**DYRK2**	**CLK1**	**CLK2**
**3a**	> 10	> 10	> 10	> 10	> 10	> 10
**3b**	> 10	> 10	> 10	> 10	> 10	> 10
**3c**	> 10	8.5	0.070	–	–	–
**5d**	> 10	> 10	0.041	0.6	0.5	7.7
